# Bell's palsy and dental procedures: A cross-sectional study

**DOI:** 10.6026/973206300220094

**Published:** 2026-01-31

**Authors:** Swet Nisha, Shashi Kumar Sinha, Debanjan Das, Sudipta Sahu, Anjali Meena, Ankita Kapoor

**Affiliations:** 1 Department of Dentistry, ESIC Medical College and Hospital, Namkum, Ranchi, Jharkhand, India; 2 Department of General Medicine, ESIC Medical College and Hospital, Namkum, Ranchi, Jharkhand, India; 3 Department of Conservative Dentistry and Endodontics, Haldia Institute of Dental Sciences and Research, Haldia, West Bengal, India; 4 Department of Oral and Maxillofacial Surgery, Haldia Institute of Dental Sciences and Research, Haldia, West Bengal, India; 5 Department of Dentistry, Lady Harding Medical College and Hospital, Delhi, India

**Keywords:** Anesthesia, Bell's palsy, dental treatment, facial nerve palsy, herpes simplex virus

## Abstract

Facial nerve palsy, including Bell's palsy, can occur as a post-dental complication, leading to sudden facial muscle weakness or paralysis. This study reviewed 2543 subjects with idiopathic facial palsy over 10 years, identifying 18 cases (0.70%) of facial paralysis post-dental treatment. All cases involved ipsilateral paralysis, with a mean onset of 3.82 days after treatment. Early diagnosis and management significantly improve prognosis, highlighting the importance of preventive measures and awareness. The identification of facial paralysis as a rare post-dental complication and the finding that early diagnosis and management significantly improve the prognosis of the condition.

## Background:

Oral health and systemic health are interconnected. Early oral health care screening and treatment can reduce disease complications and promote better patient outcomes [1]. Post-treatment complications like pain, swelling and discomfort are common following dental procedures like extraction, periodontal surgery, implant surgery and endodontic treatment [2]. However, activation of herpes simplex virus (HSV) is relatively uncommon. Few studies have reported viral reactivation of HSV as herpes labialis, with symptoms such as dull pain, burning of the lip and uneasiness being common complaints, followed by the eruption of viral exanthema [3, 4]. The etiological factors are trauma, stress and sleep deprivation. Reactivation of the virus can lead to inflammation of the facial nerve, with compression of the nerve sheath. Prophylactic antiviral medications can help to reduce the recurrence of viral reactivation. Bell's palsy has clinical features including post-auricular pain in the area of paralysis, headache, tingling sensation and inability to close the affected eyelids. Facial tone is lost in the affected area [5]. Facial nerve palsy affects the facial nerve and the muscles involved in facial expressions [6]. It is sudden in onset, can be Ipsilateral or bilateral and leads to weakness or paralysis of the affected muscle. Symptoms are associated with the muscles affected by the local anesthesia solution [7]. Common symptoms are weakness or paralysis, alteration in facial muscle expression, difficulty in closing the affected side eye, smile line alteration, drooling of saliva, face drooping, loss of facial symmetry, taste loss/alteration, difficulty in closing the affected side eye, dry eyes and irritation, tear problems and speech can become slurred. Bell's palsy is commonly seen after dental anesthetic injections, notably inferior alveolar nerve blocks [8]. Regarding the prognosis of the disease, the recovery rate is reasonable, with a high likelihood of complete recovery. Risk factors associated with viral reactivation are diabetes, immunodeficiency, stress and inflammation [9]. Post-dental procedure and reactivation of viral infection have an unknown association [10]. Local anesthetic effect can result in facial paralysis. Delayed incidence of facial palsy following dental procedure hasn't been noted [11]. Therefore, it is of interest to determining the incidence of idiopathic facial paralysis following routine dental procedures.

## Materials and Methods:

Subjects diagnosed with idiopathic facial palsy were reviewed from 2024 to 2025 in a tertiary hospital setting. The inclusion criteria were subjects who had experienced idiopathic facial paralysis within the last 10 years following dental treatment. Exclusion criteria included subjects with an unclear history of dental treatment before the onset of facial palsy, acute onset, pre-existing facial paralysis and antiviral therapy within the previous 6 months. Subjects were referred from the General Medicine department (S.K.S.) to the Dentistry department for evaluation and data recording. The data were recorded in the form of Questionnaires from the subjects. The Questions comprised of their demographic data; general medical condition; How long they were diagnosed with Facial Palsy (FP), Recovery rate, complications associated with the condition, Physical rehabilitation related to the disease, episodes of FP, Investigations associated with FP, Family history, any incidence of viral infection like herpes simplex or zoster, history of pregnancy, smoker, diabetes, Vaccination. The overall data focused on diagnosis, treatment, duration of the condition, time of recovery and outcome. We recorded subject demographics, signs and symptoms, function and treatment received (Table 1 and Table 2). Prior dental treatment procedures were recorded. The House-Brackmann Scale was used to assess facial function [11]. The study was presented and approved by the Ethical Committee. The openEpi source was used for sample size calculation based on the prevalence data from a previous study. Overview of Bell's palsy is shown is Table 1.

## Results:

A total of 2543 subjects diagnosed with facial palsy in the last 10 years were included in this study. Only 18 subjects (0.70%) were diagnosed with facial paralysis post-dental procedure. Ipsilateral paralysis was noted in all cases. Eighty-four (44%) males and 110 (55%) females were included in this study (Table 2). Only 2 subjects had presentation with systemic diseases like hypertension and diabetes mellitus. One subject was in the postpartum phase. Three subjects were smokers and had a history of alcohol use. Dental procedures performed on 18 subjects were scaling (n=7), extraction (n=3), restoration (n=3), root canal treatment (n=2) and facial infection (n=3), as shown in Table 3. Only 2 subjects received scaling and restoration in the same appointment. A total of 8 subjects received inferior alveolar block anesthesia for the treatment and rest as infiltration. No immediate facial palsy was noted post-dental treatment. After 72 hours, only three subjects experienced facial paralysis, with a mean duration of 3.82 ± 6.82 days (Table 3). Sixteen subjects reported post-auricular pain, temporomandibular joint pain, reduced taste sensation, headache, facial numbness and inability to close the affected side eyelid. Twelve subjects had received oral antiviral and steroid therapy within 4 days of the onset of facial paralysis. Tablet Prednisone 60 mg oral/day for 5 days and tablet acyclovir 1gm orally twice daily for 7 days. Six subjects received antibiotics, Specifically Amoxicillin and clavulanic acid. Three subjects didn't seek any services for one year post-incident. The recovery period was 22 days (mean) post-onset of facial paralysis. Follow-up time was 1.8 years. In the acute phase, only 6 subjects were seen; the rest were in a flaccid state. 2 subjects had mild dysfunction, while 4 patients had severe dysfunction with House Brackmann or II and III/IV, respectively. Twelve subjects experienced complete recovery within 2 to 3 months.

## Discussion:

In our study, facial paralysis following dental treatment was found to have a very low incidence, with an occurrence rate of only 0.7%. The entire 18 subjects had prodromal symptoms with viral reactivation with facial palsy on the ipsilateral side to the dental procedure. Stress during dental procedures can trigger the reactivation of viruses. Viruses like herpes simplex and varicella zoster remain dormant and can be triggered post-dental treatment [12,13- 14]. Anesthetic complications like deposition of local anesthesia near to facial nerve sheath can lead to immediate transient facial nerve palsy [15]. The management of iatrogenic facial nerve palsy can be challenging for a dentist. The signs and symptoms vary and the severity of the condition needs immediate assessment. To reduce the severity and increase the prognosis, early treatment should be started. Facial asymmetry, inability to close eyes, pain in the per-auricular area and altered taste sensation are standard features of facial nerve palsy [16]. The House-Brackmann scale is a standard clinical scale used for assessing deficient motor skills. The onset of symptoms can be immediate or delayed and recovery depends on early diagnosis and intervention. One of the iatrogenic causes is deposition of local anesthetic solution near the facial nerve sheath near the parotid gland, which can lead to transient facial palsy [17]. Dentists should be careful during local anesthetic techniques and follow landmark guidelines, considering facial nerve variations. The anesthetic solution can cause a sympathetic vascular reflex, resulting in ischemic paralysis in the region near the mastoid foramen [18]. This leads to secondary ischemic neuritis, inflammation and edema. Ramsay Hunt syndrome (facial nerve palsy and herpes zoster) was seen in only two subjects in our study. Serological assay can help in varicella zoster virus reactivation. Prolonged intraoperative times during dental procedures, such as single-sitting root canal treatment or endodontic re-treatment, can lead to nerve sheath injury. Facial nerve trauma, inflammation, or any dental procedures, as well as air blast in the extraction socket, infection, immunodeficiency, systemic conditions such as diabetes and pregnancy, can trigger the reactivation of a latent virus, causing palsy [19, 20]. Sterilization should be maintained during all dental treatment. Vascular inflammation can affect arterial blood flow, leading to ischemic nerve injury. Vaccinations like COVID-19 have a link with Bell's palsy and it could be due to similar conditions like stress and reactivation of the virus [21]. Short-duration appointments, alternative anesthetic techniques and reducing stress during appointments can help. Gentle irrigation in the extraction socket can also minimize the chances of occurrence. Prophylactic antiviral therapy and/or corticosteroids may be considered in highly susceptible cases as a preventive protocol. The recovery rate depends on early diagnosis and management. Recovery may take from months to years, depending on the severity of the condition, the patient's overall health and their treatment adherence [22, 23]. Suragimath *et al.* [24] in their case series on unilateral Bell's palsy illustrated the symptoms of palsy and similar symptoms were observed in our study as well, which helped us in making a definitive diagnosis. Dusseldorp *et al.* [25] suggested that facial reanimation surgery for Bell's palsy, particularly in cases of delayed diagnosis, can result in surgical intervention. All the subjects in our case were managed non-surgically with no reference to surgical intervention. Rourke*et al.* [26] measured the perceived emotions in Facial Paralysis subjects after post-surgical intervention and found that they had a better response towards recovery compared to non-surgical cases. Choi*et al.* [27] reported a case study on Bell's palsy following chemotherapy and radiotherapy, emphasizing the importance of early diagnosis and management of such cases through an interdisciplinary approach. This was a retrospective study with a one-year follow-up period. All subjects were managed non-surgically with an Interprofessional approach. Teamwork and collaboration are the keys to managing Bell's palsy cases. Patient awareness and education regarding the importance of seeking early care can improve the prognosis of the disease; adherence to treatment protocols can be increased through counseling and awareness about the course and outcomes of the condition. The limitations of the study were that the data were collected based on subjects' e-records and compliance with to questionnaire and the data interpretation cannot be generalized.

## Conclusion:

Early diagnosis and management can significantly improve the prognosis of the condition. Interprofessional collaboration among dentists, physicians, Ophthalmologists and physiotherapists can facilitate the holistic management of conditions, leading to better treatment outcomes. Awareness and education regarding early signs and symptoms, as well as preventive measures, among dental students can reduce the incidence of facial nerve paralysis.

## Figures and Tables

**Table 1:** Overview of Bell's palsy

**Condition**	**Clinical feature**	**Type of paralysis**	**Management**	**Management of FP**
Bell's palsy	Rapid onset (within 72 hours), ipsilateral/bilateral +/- otalgia, facial numbness	Flaccid to full recovery	Steroids +/- antivirals prednisone 60mg p.o./5 days followed by 10mg/day taper/5 days) within 72 hours. Valacyclovir, 1g p.o. twice a day/7days	Corneal protection, eye drops and eyelid stretching. Physiotherapy
Ramsay Hunt Syndrome	Ipsilateral facial paralysis, ear pain and vesicles in the auditory canal, alteration in taste perception and increased lacrimation	Flaccid to full recovery	Viral serology, (Prednisone 60 mg/5days, taper by 10 mg for the next five days) Antivirals (Valacyclovir 500 mg p.o. twice a day/ 7 days)	Corneal protection, eye drops and eyelid stretching. Physiotherapy

**Table 2:** Patients and demographics

**Demographics**	**Mean Age**	**Gender Male**	**Gender Female**	**Facial palsy Left**	**Facial palsy Right**
Bell's Palsy(n=16)	38±13.82	7	9	12	6
Ramsay Hunt Syndrome (n=2)	42±12.12	1	1	1	1

**Table 3:** Types of dental procedures leading to viral reactivation and facial palsy

Procedures	Bell's palsy (N=16)	Ramsey Hunt (N=2)	Onset FP (N=6)	Onset FP >24hrs (N=2)	FFP (N=4)	NFFP (N=2)	Mild NFFP (N=2)	Complete Recovery (N=16)
Oral Prophylaxis	6	1	2	1	2	1	1	6
Tooth extraction (+IANB)	2	1	2				1	2
Restoration (+IA)	2		1	1				2
Root canal (+IA)	3		2	1				2
Tooth abscess incision and drainage (+IA)	2				1	1		1
Re-Restoration	1				1			1
